# Biomimetic Microfluidic Pumps for Selective Oil–Water Separation

**DOI:** 10.1002/advs.202503511

**Published:** 2025-04-26

**Authors:** Zhaolong Wang, Yinfeng Li, Mingzhu Xie, Ziheng Zhan, Wenhao Li, Qihui Xie, Yong Shuai, Zhichao Dong, Zuankai Wang

**Affiliations:** ^1^ School of Energy Science and Engineering Harbin Institute of Technology Harbin 150001 P. R. China; ^2^ Zhengzhou Research Institute Harbin Institute of Technology Zhengzhou 450046 China; ^3^ Technical Institute of Physics and Chemistry Chinese Academy of Sciences Beijing 100190 P. R. China; ^4^ Department of Mechanical Engineering Hong Kong Polytechnic University Hong Kong 999077 P. R. China

**Keywords:** automatic interface capture, bionic spring microchannel, flexible microfluidics, microfluidic pump, oil–water separation

## Abstract

Addressing the challenges posed by oil pollution from both domestic and industrial sources—which contributes to energy waste and environmental degradation—is critical. Here, a new, efficient, and sustainable oil/water separation system is presented using biomimetic spring microchannels created through precise projection micro‐stereolithography‐based 3D printing technique. This innovative system allows for the swift separation of mixed oil and water phases into distinct and pure streams, achieving a high separation flux of up to 292.5 L m^−2^ h^−1^. The separation efficiency, consistently maintained over 99%, leverages the synergistic effects of surface wettability and molecular polarity to handle multiple oils with varying densities and surface tensions. Moreover, the biomimetic microchannels precise capturing of the oil/water interface and offer flexibility to initiate separation by prefilling the channels with either oil or water. Furthermore, these microchannels effectively prevent clogging, ensuring sustained performance. A significant enhancement is also demonstrated in separating crude oil from water by solar irradiation to reduce its viscosity, with a notable separation rate of 22.5 L m^−2^ h^−1^ for individual channels. The findings underscore the potential of 3D bionic functional spring microchannels for selectively separating a wide range of oil‐water mixtures with exceptional efficiency.

## Graphical abstract

A biomimetic spring microchannel system, utilizing advanced 3D printing technique, achieves swift and efficient oil‐water separation with over 99% efficiency. This innovative design leverages surface wettability and handles various oils, ensuring sustained performance and clog‐free operation for environmental remediation.

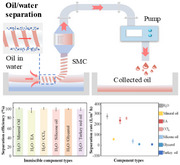

## 1 Introduction

Global energy demand is consistently increasing due to the ultra‐fast societal progress.^[^ ^1^, ^2^ ^]^ Oil, as a crucial energy resource, plays an indispensable role in powering industrial development and bolstering national economies.^[^ ^3^ ^]^ Despite its importance, the frequent occurrence of crude oil spills—caused by natural disasters, vessel collisions, and human errors—poses severe threats to the ecological environment and results in significant energy losses.^[^ ^4^, ^5^ ^]^ The current methodologies for oil recovery, including physical adsorption,^[^ ^6^, ^7^ ^]^ in situ combustion,^[^ ^8^ ^]^ and mechanical recovery,^[^ ^9^ ^]^ and others,^[^ ^10^, ^11^ ^]^ often involve complex equipment and substantial energy consumption.^[^ ^12^, ^13^ ^]^ Furthermore, these methods typically need meshes or sponges with clogging and do not allow the recovered oil to be immediately reused, underscoring the critical need for an innovative, efficient, and straightforward oil separation technology to tackle these pressing challenges.^[^ ^14^, ^15^, ^16^ ^]^ Membrane separation, characterized by micro/nanoscale pore engineering and surface wettability modulation, has demonstrated energy‐efficient and continuous oil–water separation, serving as a scalable framework for addressing these limitations.^[^ ^17^, ^18^, ^19^ ^]^

Recent advances in intelligent, stimulus‐responsive surfaces have revolutionized liquid separation technologies, offering dynamic control over wettability through pH, temperature, light, or solvent interactions.^[^ ^20^, ^21^, ^22^ ^]^ Among these, solvent‐responsive smart wetting surfaces have emerged as a particularly promising approach for selective oil‐water separation. These surfaces dynamically adjust their wettability based on molecular interactions with specific solvents, enabling precise separation of liquids with varying polarities.^[^ ^23^, ^24^ ^]^ Their ability to respond to solvent composition provides a programmable approach to liquid separation, particularly effective for emulsified oils with droplet sizes ranging from 5 to 50 µm, achieving separation efficiencies exceeding 98%.^[^ ^25^, ^26^, ^27^ ^]^ This adaptive functionality not only enhances separation precision but also addresses the limitations of conventional methods by offering scalability and reusability, paving the way for practical applications in oil/water separation.^[^ ^28^, ^29^ ^]^ However, the inherent limitations of planar surface designs in handling complex multiphase systems—such as process complexity, insufficient dynamic adaptability, and challenges in large‐scale manufacturing—have driven researchers to seek inspiration from nature's evolutionarily optimized transport architectures.^[^ ^30^, ^31^, ^32^ ^]^ This paradigm shift toward biomimetic structural engineering bridges the gap between molecular‐level responsiveness and macroscopic fluid manipulation – a critical transition that sets the stage for integrating biological design principles with advanced manufacturing technologies.

Nature provides numerous functional structures that have inspired the development of biomimetic technologies for various applications.^[^ ^33^, ^34^, ^35^ ^]^ One particularly interesting area is the study of liquid directional transport structures, which are increasingly recognized for their practicality across multiple fields such as fog collection,^[^ ^36^, ^37^ ^]^ seawater desalination,^[^ ^38^, ^39^ ^]^ microreaction,^[^ ^32^, ^40^ ^]^ and oil‐water separation.^[^ ^30^, ^41^ ^]^ For instance, the directional transport of microdroplets on cactus spines exemplifies this, where unique hierarchical and trichome architectures on the stems enhance liquid movement in arid climates.^[^ ^37^, ^42^ ^]^ Structural innovations in oil/water separation devices have garnered significant research attention, particularly through bioinspired engineering strategies.^[^ ^43^, ^44^, ^45^ ^]^ Exemplifying this trend, grooved conical spine arrays, architected through biomimetic integration of fishbone morphology and rice leaf venation patterns, achieve unprecedented underwater oil transport velocity via precision‐engineered multi‐bioinspired grooved conical spine fabricated by 3D printing technologies.^[^ ^46^ ^]^ Furthermore, energy‐autonomous separation systems employing capillary‐siphon hybrid mechanisms demonstrate revolutionary performance: anti‐gravity fluidic pumps engineered with hierarchical microchannels spontaneously segregate purified oil/water phases from emulsions without external energy input.^[^ ^47^ ^]^ Most strikingly, dual‐bioinspired overflow systems mimicking the *Nepenthes* peristome and cat tongue surface architectures enable phase‐pure separation of oil‐in‐water emulsions through synergistic liquid steering effects and directional overflow control.^[^ ^30^ ^]^ These structural breakthroughs collectively redefine the paradigm of intelligent separation technologies by harmonizing nature inspired designs with precision manufacturing capabilities.

Here, drawing inspiration from the morphometric characteristics of cucumber tendril springs, we have demonstrated a biomimetic spring microchannel (SMC) utilizing the projection micro‐stereolithography (PµSL) based 3D printing technique to achieve flexibility, stretchability, and efficient liquid transport, enabling the seamless and rapid separation of oil and water. The proposed biomimetic SMCs, endowed with continuous helical microstructures, promote the directional flow and separation of liquids, and selectively prevent the ingress of other liquids and gases, capitalizing on their varying molecular polarities. The spring‐like design enhances oil–water separation efficiency through dynamic deformation, offering superior adaptability to various viscosities and flow rates, while its open structure prevents clogging and ensures sustainable performance, complemented by a modular microchannel design for scalable industrial integration. This straightforward yet highly effective and precise 3D printing approach yields microfluidic constructs that are both scalable and boast exceptional separation efficiency.^[^ ^48^, ^49^, ^50^ ^]^ The biomimetic SMCs emerge as an innovative solution for liquid separation, achieving an impressive 99% separation efficiency at a peak rate of 292.5 L m^−2^ h^−1^. It also demonstrates remarkable efficiency in separating high‐viscosity fluids such as glycerol and petroleum. Notably, these bionic microchannels were effectively employed to separate crude oil that was heated by solar energy, thereby reducing its viscosity and showcasing their potential for practical applications.

## 2 Results and Discussion

**Figure 1:**
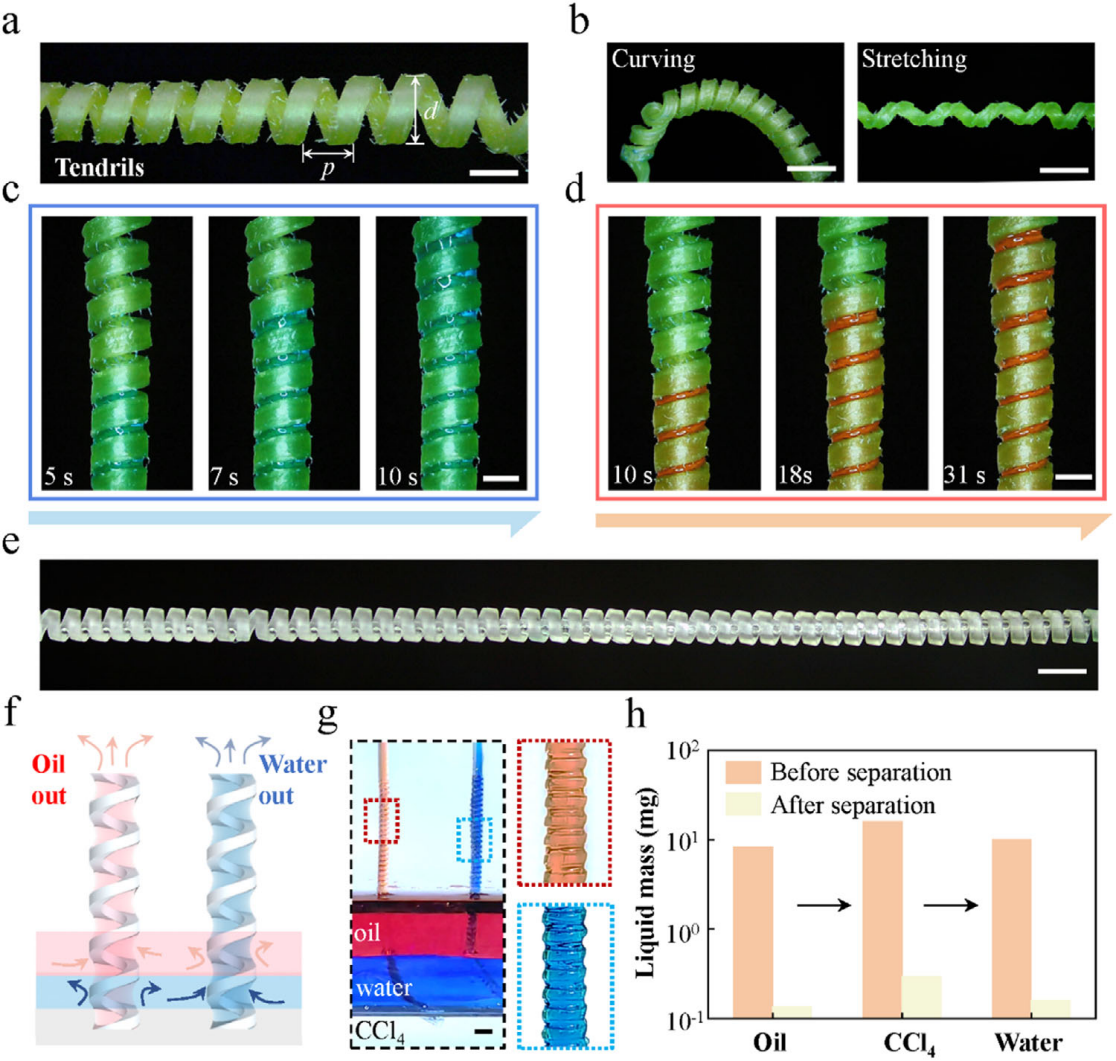
Structural characteristics of cucumber tendrils for oil‐water separation. a) Optical image of a cucumber tendril displaying the helical morphology of the coiled tendril. The pitch (*p*) and diameter (*d*) of the tendril are ≈1.5 and ≈2 mm, respectively. b) Bending and stretching properties of tendrils. Scale bar, 2 mm. c,d) Transport of water and oil with cucumber tendrils. Water is transported along the tendril at a higher speed, whereas oil is transported along the tendril at a lower speed. Scale bar, 1 mm. e) Optical image of a biomimetic SMC. Scale bar, 1 mm. f,g) Sketch and optical images of the selective separation of individual liquids by placing SMCs prefilled with water or oil onto an oil–water–CCl_4_ mixture. Scale bar, 500 µm. h) Mass change in the oil–water–CCl_4_ mixtures before and after SMC separation.

**Figure** **1**a shows the morphology of the cucumber plant tendrils, which are characterized by spiral threads of varying morphologies and geometric parameters, arranged longitudinally at intervals. Each tendril has a diameter (*d*) of ≈2 mm and a pitch (*p*) of ≈1.5 mm (Figure S1, Supporting Information). These tendrils have evolved remarkable flexibility, enabling them to maintain a high degree of elasticity. They can revert to their original form after being bent or stretched (Figure 1b; Figure S2, Supporting Information). The tendrils exhibit distinct hydrophilic and lipophilic properties, with water and oil contact angles measured at ≈60° and 15°, respectively (Figure S3, Supporting Information). Intriguingly, when water and mineral oil are separately applied to the base of a vertically positioned cucumber tendril (Video S1, Supporting Information), both liquids ascend, with water moving faster than oil (Figure 1c,d).

Motivated by these observations, we applied 3D printing technology (Figure S4, Supporting Information) to create biomimetic SMCs with rectangular cross‐sectional shapes and varying structural parameters like diameter and pitch (Figure 1e; Figure S5, Supporting Information). The different wettability and molecular polarities of water and oil facilitate a unique separation mechanism (Figure S6, Supporting Information): water prefills the SMCs, effectively blocking oil from entering the microchannels, as evidenced in Figure S7 (Supporting Information). Conversely, when the SMCs are prefilled with oil, water is similarly obstructed, allowing the oil within the microchannels to flow undisturbed by water. This selective permeability can enhance the functionality of the biomimetic SMCs in fluid management and separation applications, which we will discuss in the following.

Based on the flexibility, stretchability, and liquid transport performance characteristics of SMCs with specific liquids in multi‐component systems, we developed a selective SMC pump‐based liquid separation device. When SMCs, which are initially filled with water, are positioned at the oil–water–CCl_4_ mixture, the middle water can be selectively separated and removed, independent of the overlying oil layer (Figure 1f,g). Similarly, when SMCs are prefilled with oil and positioned into the oil–water–CCl_4_ mixture, an upward pumping action allows for the separation and extraction of the top oil layer. As the upper oil layer is disengaged, the separation system continues to isolate the lower CCl_4_ phase, enabling the selectively sequential separation of the three fluid components. The mass changes of the mixture of oil–water–CCl_4_ mixture before and after separation are shown in Figure 1h. After separation, the mass differences before and after the separation of the oil, the CCl_4_, and the water are 0.14, 0.3, and 0.16 mg, respectively.

**Figure 2:**
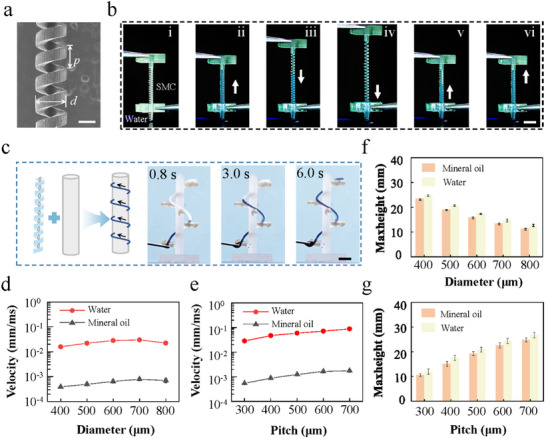
Microfluidic performance of the SMCs. a) SEM image of a SMC. Its diameter *d* and pitch *p* are 200 and 500 µm, respectively. b) Control of the maximum liquid transport height by stretching a SMC. Scale bar, 2 mm. c) Capillary rise of liquid inside a SMC along a spiral path. Scale bar, 5 mm. d,e) The average capillary rise velocities of the water and oil inside the SMCs with different geometric parameters. f,g) Maximum heights of the water and oil inside the SMCs with different geometric parameters.

Furthermore, we implemented a 3D printing technique to fabricate the SMCs. The cross‐sectional shapes of our SMCs are rectangular with different structural parameters (diameter (*d*) and pitch (*p*), **Figure** **2**a). During the stretching of an SMC, when the bottom of the SMC contacts the liquid, the liquid is rapidly transported to the apex. When we stretch the SMC two times longer than its original length, the liquid inside the SMC gradually descends and becomes flush with the bottom. Subsequently, upon withdrawal of the applied force, the SMC gradually contracts, and the liquid is transported back to the top under the action of capillary force (Figure 2b; Video S2, Supporting Information). Throughout this process, the elongation of an SMC is controlled to regulate the maximum height of the fluid, thereby preventing overflow (Figure S8, Supporting Information). We then tested the flexible fluid transport performance attributes of SMCs with arbitrary shapes (Figure 2c). The SMCs exhibit good flexibility without affecting their microfluidic performance (Video S3, Supporting Information).

We subjected the vertically oriented SMCs to water and oil at sufficiently slow speeds. The transport velocities of water and oil inside the SMCs first increase and then decrease as their diameter (*d*) increases from 400 to 800 µm. The transport velocities of water and oil both occur at *d* = 700 µm, which are 0.03 and 0.0007 m s^−1^, respectively (Figure 2d). The maximum capillary rise height (*h*) of water in the SMCs decreases from 25 to 10 mm, while the maximum capillary rise height (*h*) of oil in the SMC decreases from 18 to 5 mm (Figure 2f). Additionally, we keep the other structural parameters of the SMC constant but increase the pitch (*p*) of the SMCs from 300 to 700 µm, and the water and oil transport velocities gradually increase with increasing pitch to values reaching 0.09 and 0.002 m s^−1^, respectively (Figure 2e). The *h* of water inside the SMCs increases from 15 to 30 mm, while the *h* of oil inside the SMCs increases from 8 to 21 mm (Figure 2g). The *h*–*t* diagrams of water transport in SMCs with different structural parameters are shown in Figure S9 (Supporting Information).

**Figure 3:**
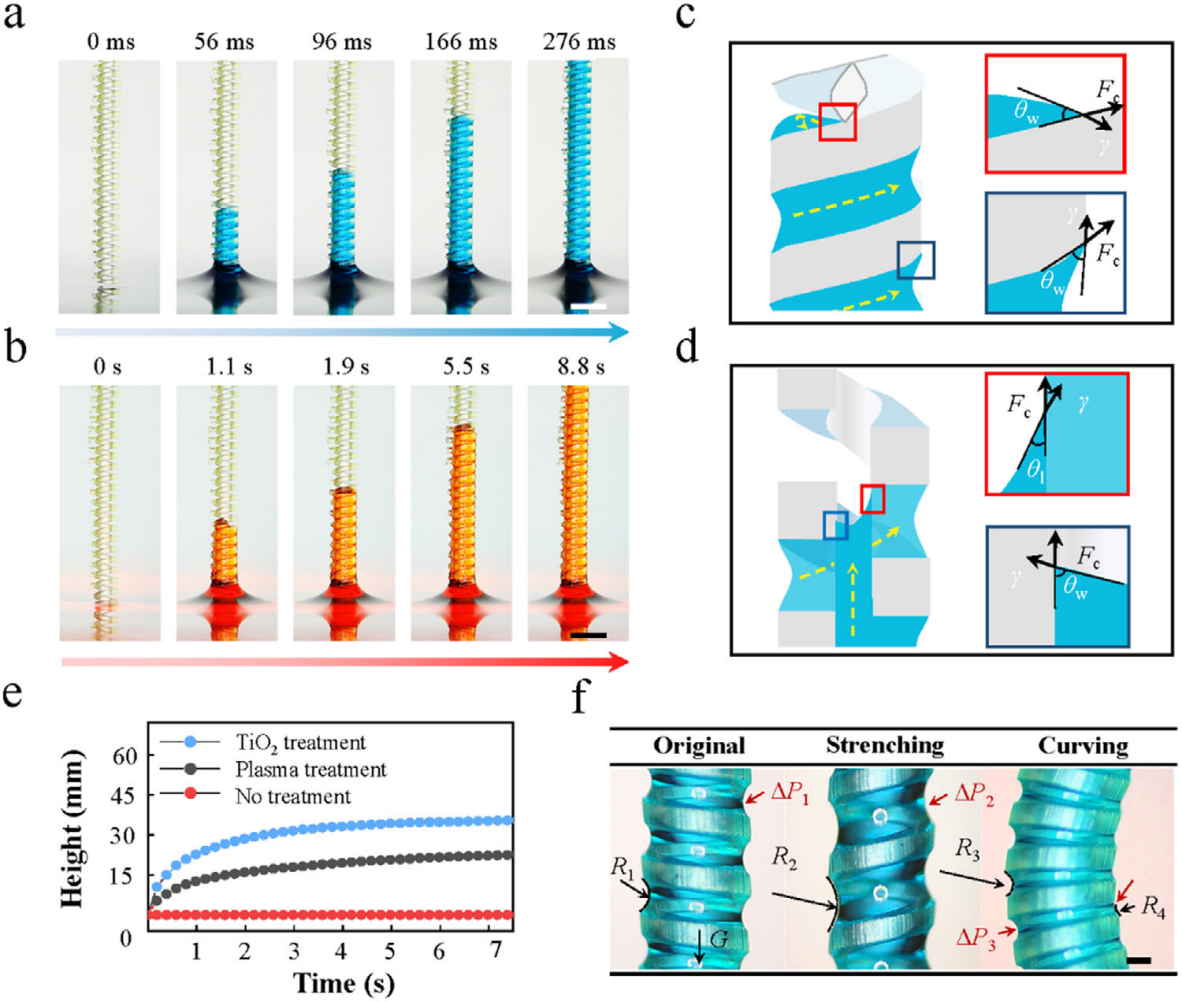
Mechanisms for liquid transport in the SMCs. a,b) Transporting phenomena of different liquids in SMCs, and the time of water transport within a given distance is significantly shorter than that of oil. Scale bar, 500 µm. c) Liquid flows along inclined surfaces, which is driven by capillary force between two inclined surfaces. d) Liquid flows along the inner cylinder space composed of the wall surface and the superhydrophilic water–film interface. e) Maximum height of a liquid for various solid–liquid contact angles resulting from different surface treatments. f) Analysis of the dynamic processes and equilibrium states exhibited by SMCs with different morphologies. Scale bar, 100 µm.

We further investigated the water and oil microfluidic characteristics of SMCs for liquid transport (**Figure** **3**a,b; Video S4, Supporting Information) with detailed force analyses for two representative scenarios involving inclined surfaces and inner wall surfaces. For the inclined surface rising scenario (Figure 3c), liquid spiraling climbs upward (Video S5, Supporting Information). There is a solid‐liquid contact angle of *θ*_w_ at the inclined surface of the SMC, and this action is driven by the capillary force *F*_c_ between two inclined surfaces. Regarding the inner wall surfaces (Figure 3d), after the liquid undergoes a capillary rise phenomenon along the two inclined surfaces and fills the space between them, the liquid and the inner wall surface form a new capillary flow section. This section rises vertically under the action of capillary force. Due to the superhydrophilicity at the water‐film interface (liquid‐liquid contact angle *θ*_l_), where the liquid is subjected to tensile forces, the liquid rise is slightly delayed compared to the movement of the liquid on an inclined surface. For different solid–liquid contact angles *θ*_w_ caused by different surface wettability, the corresponding maximum heights of the fluid also differ from each other, and the fluid treated with the plasma has the highest height of 30 mm (Figure 3e; Figure S10, Supporting Information).

In the process of capillary rise within the SMCs, a meniscus forms between the two inclined surfaces, characterized by a specific meniscus radius (*R*_1_) and a corresponding Laplace pressure difference (Δ*P*_1_), as illustrated in Figure 3f and further detailed in Figure S11 (Supporting Information). As the pitch (*P*) of the SMC increases due to elongation, there is an increase in the meniscus radius to *R*_2_, resulting in a decreased Laplace pressure difference (Δ*P*_2_ <Δ*P*_1_). This indicates a direct relationship between pitch elongation and the dynamic changes in fluid mechanics within the SMC. Furthermore, the bending of the SMC creates an asymmetry in pitch distribution—reducing on the curved side and increasing on the opposite. This alteration affects the meniscus radii (*R*_3_ and *R*_4_), with *R*_4_ decreasing near the bend and *R*_3_ increasing away from the bend. Consequently, this leads to an asymmetric distribution of capillary forces, which in turn affects the Laplace pressure difference across these regions.

The initial height of the liquid prefilling layer can be precisely controlled by adjusting the morphologies of the SMCs before initiating the selective separation process. Additionally, the inherent flexibility of the SMCs along with external forces during separation plays crucial roles in influencing the internal liquid flow dynamics.

**Figure 4:**
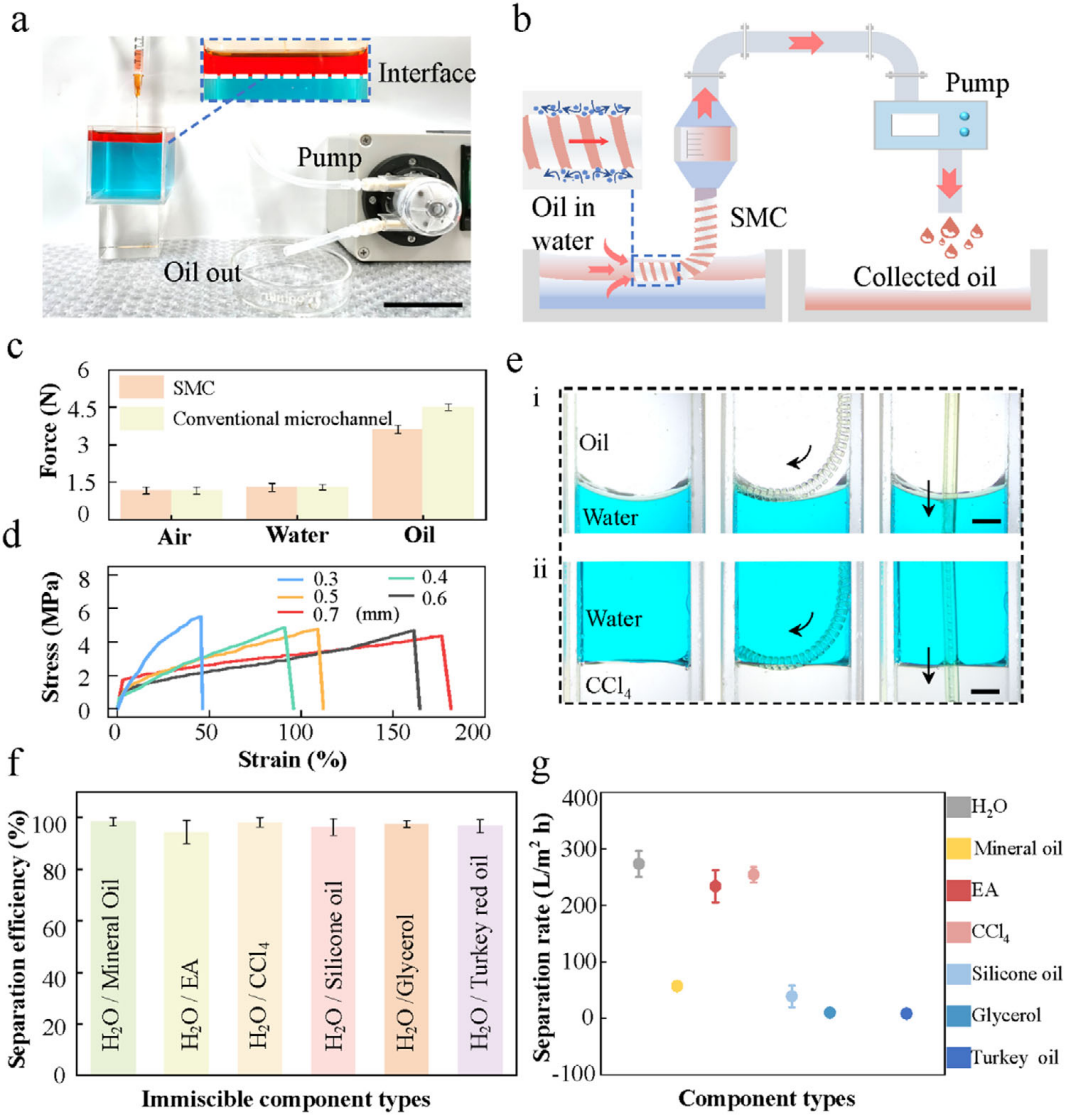
SMC system for oil/water separation. a,b) Schematic and optical images of the assemblies for oil/water separation. A SMC is filled with oil and floats on the oil/water interface, and the pump removes the oil from the mixing system. Scale bar, 5 cm. c) Comparison of the pulling forces required for SMCs and ordinary microchannels for different types of liquid transport. d) Stress–strain diagram of SMCs in the wetted state. e) Automatic capturing of the water/oil interfaces for the SMC in water/oil mixture (left: SMCs; right: ordinary microchannels). Scale bar, 2 mm. f) Separation efficiencies of two immiscible components. g) Maximum separation fluxes of different types of liquids through SMCs.

Our investigations extend to the separation characteristics of two immiscible liquid components within these bionic SMC structures (**Figure** **4**a,b). The forces influencing the liquid dynamics during separation can be categorized into four primary groups: i) pump pressure, ii) liquid surface tension, iii) gravity, and iv) adhesive forces, detailed in Figure S12 (Supporting Information). Notably, the surface tension of the liquid, which varies with the type of liquid, plays a critical role. As depicted in Figure S12‐i (Supporting Information), if the aggregate force on either side of the SMC does not exceed the Laplace pressure difference caused by the concave menisci, the fluid will be efficiently transported without the intrusion of air or other fluids. Conversely, if the combined force acting on the fluid on each side of the SMCs is greater than the Laplace pressure difference due to the concave surface of the liquid, then the liquid column will crack or break (Video S6, Supporting Information). Thus, air or other liquids are allowed to enter and reduce the separation efficiency (Figure S12‐ii,iv, Supporting Information).

Figure 4c illustrates the required pulling force to pump an equivalent volume of liquid through an SMC and a conventional microchannel of the same diameter. When the medium's viscosity is low, such as air and water, the pulling force difference between the SMCs and the conventional microchannels remains minimal. However, for high‐viscosity liquids like oil, the SMCs significantly reduce liquid resistance during movement, resulting in a 30% decrease in pulling force compared to the conventional microchannels. Consequently, the SMCs separation system demonstrates enhanced energy efficiency in liquid transport over traditional microchannels. Furthermore, we evaluated the stress–strain characteristics of SMCs in both wet and dry states, as depicted in Figure 4d and Figure S13 (Supporting Information). The findings reveal a gradual decline in stress–strain performance as the diameter of SMCs increases. Specifically, for SMCs with diameters exceeding 500 µm, the fracture stresses are substantially higher in wet conditions than in dry one. This disparity diminishes in SMCs with diameters below 500 µm, where the fracture stresses of wet and dry conditions show negligible differences. Additionally, SMCs with a diameter of 700 µm exhibit markedly improved stretchability under wet conditions compared to dry one. In contrast, for diameters smaller than 700 µm, the strain differences under the two conditions are minimal.

The flexibility and stretchability of SMCs underpin the mechanisms of selective oil–water separation in SMC systems. These systems excel in automatically capturing interfaces between incompatible liquids (Video S7, Supporting Information), enhancing operational efficiency. Unlike traditional microchannels, SMC systems do not require monitoring of the oil–water interface, significantly reducing the risk of cross‐contamination from other liquids. This is depicted in Figure 4e‐i, which shows an oil–water immiscible solution, and Figure 4e‐ii, illustrateic in a CCl_4_‐water immiscible solution. In these figures, SMCs accurately capture both oil–water and water‐CCl_4_ interfaces. In contrast, conventional microchannels, which intersect these interfaces, necessitate continual adjustment to avoid unintended liquid intake during separation. Our experiments also show the successful separation of glycerol and turkey red oil from water. As illustrated in Figure 4f, SMCs achieve separation efficiencies of ≈99% across various immiscible liquids.

Moreover, we assessed the maximum separation rates in SMCs without air bubbles, as shown in Figure 4g. The water exhibits the highest separation rate at 292.5 L m^−2^ h^−1^, while the more viscous turkey red oil shows the lowest rate at 8.57 L m^−2^ h^−1^. Figure S14 (Supporting Information) further explores the wettability of fabricated surfaces with different liquids, demonstrating that liquids with higher surface tension and density (e.g., water and ethyl acetate (EA)) achieve greater maximum transport flux, whereas those with lower surface tension and density (e.g., glycerin and turkey red oil) exhibit lower fluxes.

**Figure 5:**
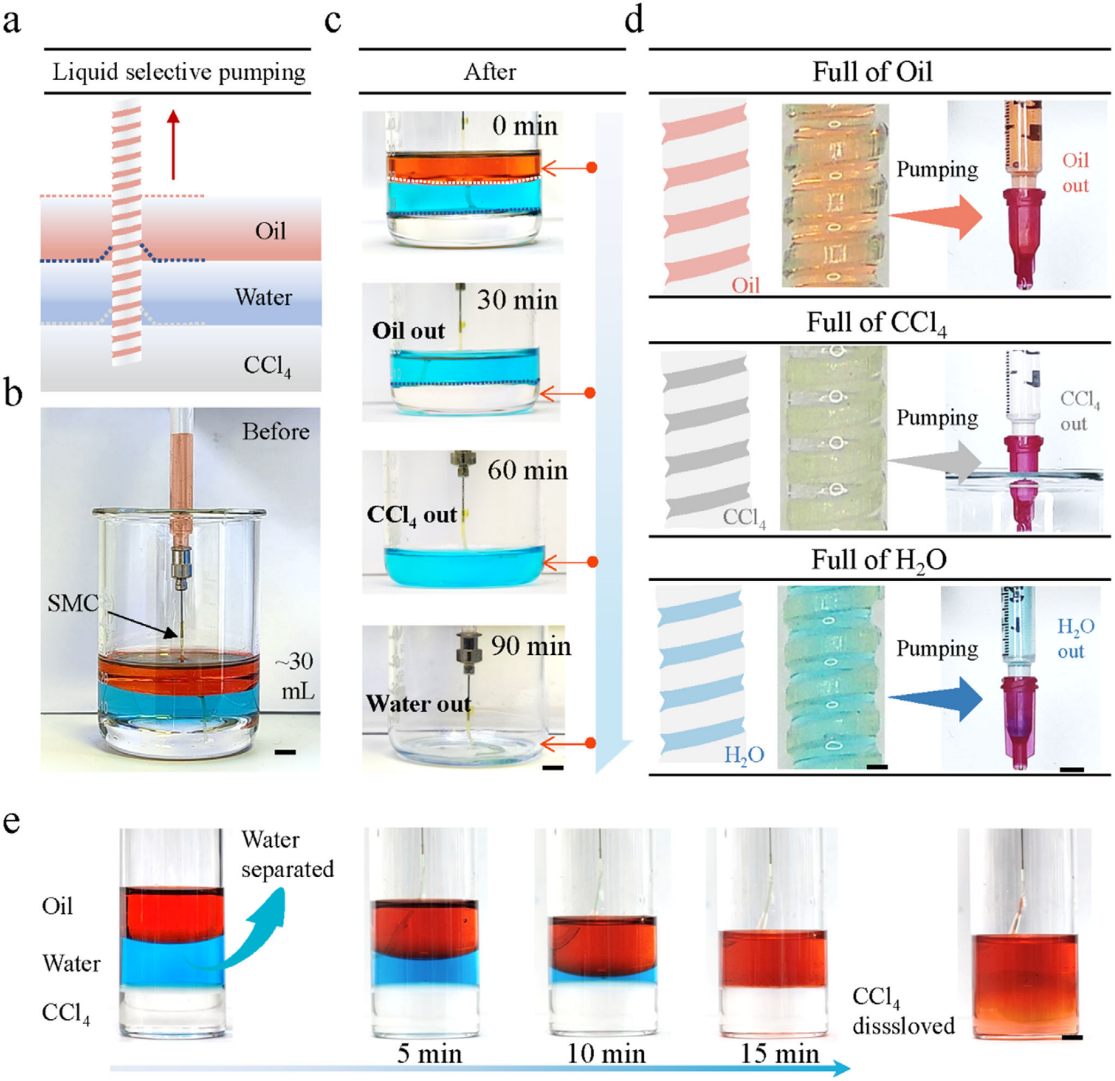
The SMC system for the selective separation of multilayered immiscible components. a,b) Experimental assembly of the SMC system for multilayered immiscible components separation. From top to bottom, the three liquids are oil, water, and carbon tetrachloride. Scale bar, 1 cm. c) Experimental separation of mineral oil, carbon tetrachloride, and water in sequence. Scale bar = 1 cm. d) Schematic and optical images of fluids and fluids collected from SMCs during the separation process. (left, scale bar = 200 µm; right, scale bar = 1 cm). e) Application of SMC separation systems as microreactors. The microvessel consists of three immiscible liquids, with the upper and lower layers capable of reacting with each other. The intermediate medium acts as a barrier between them. The SMC separation system effectively separates the intermediate medium, allowing the upper and lower layers to react. Scale bar, 1 cm.

Previous studies focused on the separation of two immiscible components.^[^ ^51^, ^52^, ^53^ ^]^ However, immiscible mixtures with more than two components are common in both industrial and domestic wastewater. Therefore, we tested the simultaneous separation of multiple mutually incompatible liquids based on our SMC separation device. Ten milliliters of CCl_4_, water, and oil were successively injected into a vessel (**Figure** **5**a,b). Subsequently, a SMC is prefilled with oil and immersed in an oil–water–CCl_4_ three‐layer mixture for separation. The top layer of oil is separated by the SMC within 30 min. However, the intermediate layer of water is rejected, and it never enters the SMC due to the oil film that forms at the opening of the microchannel. Afterward, the SMC separates the bottom CCl_4_ layer through the water within another 30 min. Thus, liquids can continuously travel upward along the SMC across the oil‐water interface or water‐CCl_4_ interface, as shown in Figure 5c and Video S8 (Supporting Information). Figure 5d shows schematic and detailed images of the SMC in the gradual separation of the multilayered liquid, which is transported upward along the SMC and separated individually. This approach offers a solution for liquid extraction from complex multiphase fluid environments.

In addition, the liquid‐selective separation properties of SMC can be effectively used to create a reaction site for time‐space controllable liquid‐phase chemical reactions. Figure 5e and Video S9 (Supporting Information) show an example where a vessel is filled with a three‐immiscible‐liquid system consisting of oil, water, and CCl_4_. The oil and CCl_4_ in the top and bottom layers are able to dissolve with each other, while the water in the middle layer does not mix with the other two liquids. The water acts as an insulator, creating a barrier between the oil and CCl_4_. By utilizing the selective liquid separation properties of SMC, the water in the middle layer can be separated from the system. This separation process allows the oil and CCl_4_ to come into contact and dissolve, initiating the desired chemical reaction. By controlling the volume of intermediate liquid (water) according to the separation rate of the SMC, the reaction delay time can be precisely controlled. In addition, the size of the vessel can also be controlled, further enabling time‐space control of the liquid phase chemical reaction. The selective separation performance of the SMC offers significant potential for a wide range of applications in controlled chemical reactions, microreactors, and bioengineering. It enables precise control of reaction timing and provides a platform for designing and performing reactions in specific time‐space configurations.

**Figure 6:**
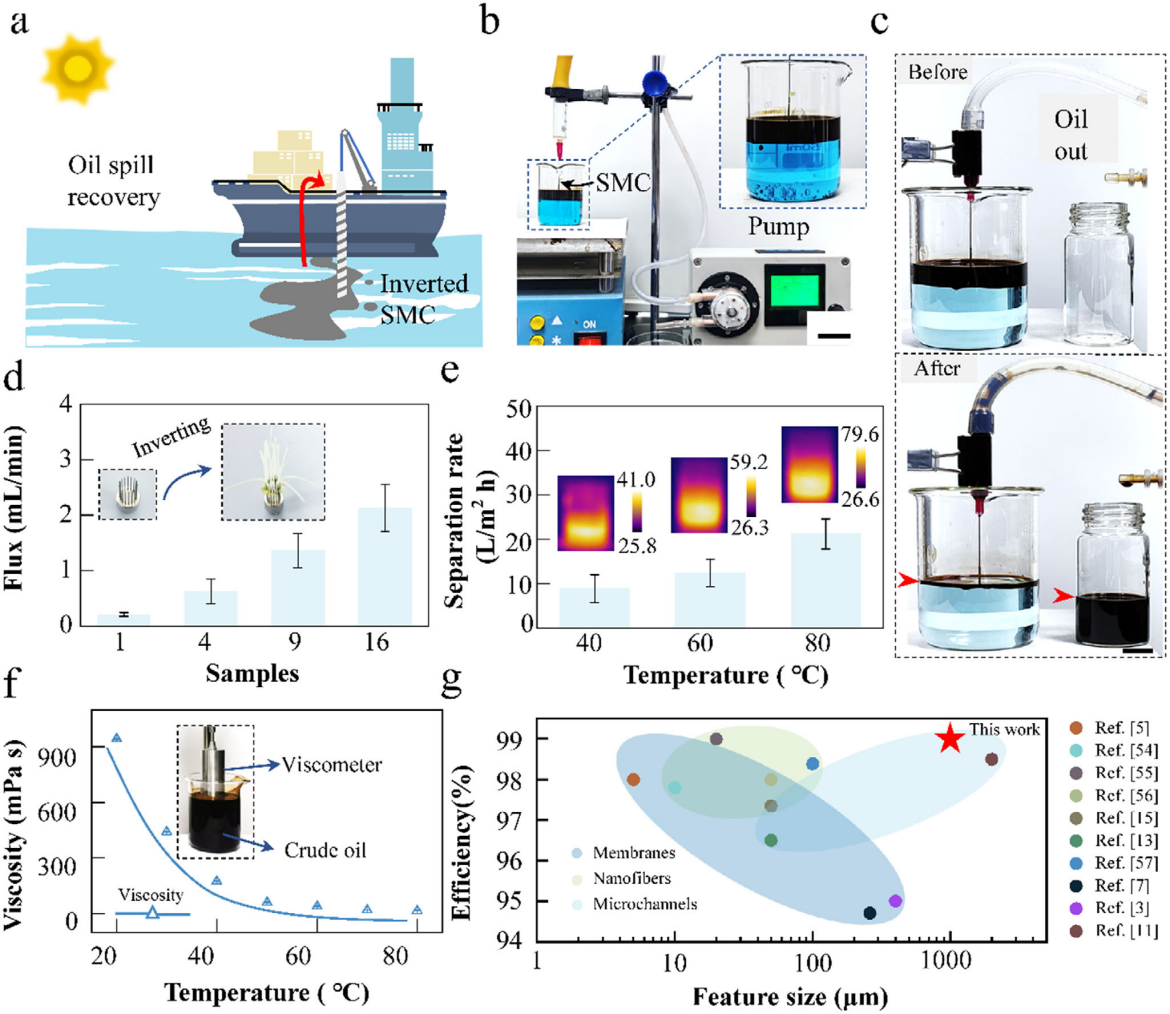
Crude oil–water separation performance of our SMC system. a) Schematic of the SMC system for crude oil–water separation. Viscous oil floats on the sea, and the oil is pumped through our bionic SMC system into the container. b) Experimental setup of the SMC separation system. The blue color represents water. The black color represents the crude oil that floats on water. Crude oil is pumped from the left chamber to the right chamber. Scale bar, 3 cm. c) Optical images of the crude oil separation process with our bionic SMCs at 40 °C. Scale bar, 1 cm. d) The crude oil separation rate of multiple SMCs in the separation system. e) The separation rate of the SMC separation system at different temperatures. f) The evolution of the crude oil viscosity at different temperatures. g) Diagram summarizing the relationship between the feature size and separation efficiency of different separation methods, including microchannels, membranes, and nanofibers. The dots in the bluish circle and the bluish dots represent microchannel separation. The dots in the green circle and the green dots represent membrane separation. The dots in the sky‐blue circle and the sky‐blue dots represent microstructure separation.

Oil spills in marine environments not only pose significant environmental risks but also lead to substantial economic damages. Our research utilizes bionic SMCs to address these challenges through efficient and sustainable oil–water separation. As demonstrated in **Figure** **6**a,b, we tested the potential applications of SMC for water‐oil seperation, who is 700 µm in diameter and 200 µm in depth with a pitch of 700 µm. To enhance the separation process, we simulate the effects of solar heating on crude oil, as shown in Figure 6f. The heating effectively reduces the viscosity of the oil, increases its transport velocity through a SMC. The method involves vertically inserting the SMC into oil‐contaminated water and pumping the heated crude oil from the surface to where it is collected, as recorded by a digital camera (Figure 6c). The SMCs efficiently transfer the crude oil from the left to the right side of the setup, achieving a collection rate up to 22.5 L m^−2^ h^−1^, as detailed in Figure S15 and Video S10 (Supporting Information). Expanding on single SMC capabilities, we explore large‐scale crude oil separation using arrays of 4, 9, and 16 SMCs, as depicted in Figure 6d and Figure S16 (Supporting Information). The simultaneous operation of multiple SMCs substantially increases the separation rate with a configuration of 16 SMCs reaching a flux of 2.13 mL min^−1^, nearly ten times that of a single SMC, and maintaining over 99% separation efficiency. However, the performance of crude oil–water separation is significantly influenced by ambient temperature, which affects the efficiency of the solar‐heated process.

To mitigate the effects of varying natural solar radiation intensities, we conducted a series of tests to determine the maximum separation rates at different temperatures, as illustrated in Figure 6e. The results indicate that the separation rate of crude oil is 9.6 L m^−2^ h^−1^ at 40 °C, while it is 22.5 L m^−2^ h^−1^ at 80 °C. Specifically, the crude oil separation rate increases proportionally with increasing temperature because the viscosity of the crude oil is greatly affected by its temperature. As indicated in Figure 6f, the viscosity of crude oil is 943.5 mPa·s at 20 °C, who decreases to 16.6 mPa·s at 80 °C, confirming that the temperature of the crude oil critically contributes to its flow characteristics. The summary diagram shows the obvious advantages of our SMC over other liquid separation devices,^[^ ^54^, ^55^, ^56^, ^57^ ^]^ including oil/water separation devices based on microchannels, membranes, and microstructures (Figure 6g).

## 3 Conclusion

In summary, our research introduced and developed biomimetic spring microchannels utilizing the PµSL 3D printing technique, meticulously replicating the intricate morphology of cucumber tendrils. This innovation has successfully achieved highly efficient oil–water separation rates over 99%. The separation process leverages a synergistic mechanism, combining surface wettability and molecular polarities to facilitate a seamless, continuous, and efficient separation without any decline in flux. Moreover, the concurrent operation of multiple microchannels significantly boosts the separation throughput, making it suitable for large‐scale applications. These biomimetic SMCs hold considerable promise for use in diverse sectors such as sewage treatment, ocean governance, and environmental protection, as corroborated by our validations.

## 4 Experimental Section

### Natural Materials

Cucumber tendrils were obtained at Changsha, Hunan Province. It consists of spiral threads with different morphologies and geometric parameters, which were arranged longitudinally at intervals. The diameter (*d*) and pitch (*p*) of each tendril were ≈2 and ≈1.5 mm, respectively.

### Characterization of the Cucumber Tendrils

Optical images of the cucumber tendrils were recorded using a 4K HD camera (WST‐4KCH). The structure of SMCs was observed using SEM (MIRA4 LMH, TESCAN, China) at an accelerating voltage of 20 kV. To avoid severe deformation caused by dehydration, a freeze‐drying method was used to prepare the SEM specimens.

### Observation of Liquid Transport

The vertical transport of liquids across SMCs was investigated by analyzing the characteristics of capillary rise. The test liquids were deionized (DI) water and mineral oil, which were placed in discs at a depth of 10 mm. First, the SMCs were placed in a vertical upward position and inserted into DI water to promote capillary rise. The dynamic process of capillary rise was recorded by a high‐speed camera (Memrecam HX‐7 s, NAC, Japan). The contact process between the DI water and the bionic helicoidally patterned microchannel was filmed (1000 fps). Then, Tracker software 4.9.1 was used to record the meniscus height at various time points with every six frame intervals starting from zero, which enabled plotting of the relationship between the filling height “*h*” and filling time “*t*.”

### Fabrication of Bionic Spring Microchannels

The 3D model of bionic SMCs was constructed using SolidWorks and then sliced into a series of 2D images with a layer thickness of 5 µm using specific slicing software. Then, a PµSL‐based 3D printer (BMF S140, Shenzhen, China) was used to fabricate all 3D complex microstructures layer‐by‐layer with a resolution of 10 µm × 10 µm by controlling the exposure time to UV light (405 nm) to expose the liquid photosensitive resin. The printed SMCs were removed from the printing platform, immersed in an ethanol solution, and ultrasonically cleaned for 1 min to wash off the uncured photosensitive resin on their surfaces. The SMCs were printed with a layer thickness of 5 µm and an exposure time of 2 s. In comparison, the unstructured substrate was printed with a thickness per layer of 40 µm and an exposure time of 5 s to enhance the printing efficiency.

### Contact Angle Measurements

The CAs of the printed structured surfaces were measured using a CA measurement device (SDC‐100, SINDIN Company, China). The bionic structured surface was placed on a test platform. Then, a 2 µL DI water droplet was ejected from a syringe needle on the bionic structured surface. The image acquisition system transmitted the side view of the water droplet placed on the surface to specific software (SDC‐100V3.1.2) to calculate the CA.

### Oil–Water Separation Test

The oil–water separation capability of the SMCs was investigated using a mixture of crude oil (10 mL) and DI water (30 mL). The oil–water mixture was preheated on a heating stage (NBW‐2020, Lambert, China) and subsequently irradiated under a solar simulator (CEL‐S500, Ceaulight, China) with an intensity of 1 kW m^−2^. The SMC was vertically immersed into the mixture, enabling the spontaneous rising of crude oil into its opening structure via capillary forces. Selective extraction of crude oil was accomplished by operating a peristaltic pump (LH006L, Lianhezhongwei Technology Co., Ltd., China) at maximum separation velocity (Δ*p* = *σ*/*G*), achieving efficient phase separation within the biphasic system.

### Separation Efficiency Calculation

The initial mass of the oil–water mixture (*m*_a1_) and the mass of the crude oil (*m*_o_) were measured using an electronic balance (AS. R, Radwag Inc., Switzerland) prior to the separation process. The crude oil was then separated using the SMC system. Afterward, the final weight of the oil–water mixture (*m*_a2_) was measured. The separation efficiency (*e*) is calculated as follows:

(1) $$e=\left[\left(m_{a1}-m_{a2}\right)/m_{o}\right]\times 100\%$$

Additionally, the oil–water separation rate (*v*) is determined through quantitative analysis of the separation process. Time‐lapse photography was employed to record the total separation duration (*t*), while the cross‐sectional area (*S*) of the SMC and the collected crude oil volume (*V*) were precisely measured. The separation rate is subsequently calculated using the formula

(2) v=V/(t·S)

### Fluid Viscosity Test

A heating plate was employed to heat the crude oil and a thermocouple to measure the oil temperature. The viscosities of the crude oils were characterized using a rotational viscometer (NDJ‐5S, YiXin, China). First, a suitable rotor was selected for the viscosity measurement based on the viscosity range of the crude oil at a specific temperature. The lifting frame of the viscometer was rotated to gradually lower the viscometer, allowing the rotor to be immersed in the crude oil until the designated mark on the rotor was located at the liquid surface. Prior to the measurement, the rotor was immersed in the crude oil for 3 min to ensure temperature equilibrium between the rotor and the crude oil. Then, the viscosity of the crude oil was recorded after the rotor rotated 20 times in the liquid. This procedure was repeated five times for each data set to ensure reproducibility.

### Statistical Analysis

For the liquid transport and oil–water separation performance measurements, at least five duplicate cells were repeated to ensure the validity. The data deviation was typically within ≈3% and the reported values were the mean ± standard deviation (SD). The Origin software was used for data analysis and processing.

## Conflict of Interest

The authors declare no conflict of interest.

## Author Contributions

Z.W. and Z.D. conceived the project, and Y.L. carried out the experiments and analyzed the data, and M.X., W.L., and Q.X helped to analyze the data. Z.W. and Y.L. wrote the manuscript, and Z.W. and Z.D. revised the whole manuscript. Y.S. provided the methodology and investigation. Z.W., Y.S., Z.D., and Z.W. supervised the whole project. All the authors discussed the results and commented on the manuscript.

## Supporting information



Supporting Information

Supplemental Video 1

Supplemental Video 2

Supplemental Video 3

Supplemental Video 4

Supplemental Video 5

Supplemental Video 6

Supplemental Video 7

Supplemental Video 8

Supplemental Video 9

Supplemental Video 10

## Data Availability

The data that support the findings of this study are available in the supplementary material of this article.

## References

[1] J. R. Fanchi , Energy in the 21st Century: Energy In Transition, World Scientific, Singapore 2023.

[2] Y. Guan , J. Yan , Y. Shan , Y. Zhou , Y. Hang , R. Li , Y. Liu , B. Liu , Q. Nie , B. Bruckner , K. Feng , K. Hubacek , Nat. Energy 2023, 8, 304.

[3] Y. Shi , W. Yang , X. Feng , Y. Wang , G. Yue , S. Jin , Appl. Surf. Sci. 2016, 367, 499.

[4] A. Jernelöv , Nature 2010, 466, 182. 20613822 10.1038/466182a

[5] J. P. Chaudhary , S. K. Nataraj , A. Gogda , R. Meena , Green Chem. 2014, 16, 4552.

[6] T. Ono , T. Sugimoto , S. Shinkai , K. Sada , Nat. Mater. 2007, 6, 429. 17468762 10.1038/nmat1904

[7] L. Li , Z. Liu , Q. Zhang , C. Meng , T. Zhang , J. Zhai , J. Mater. Chem. A 2015, 3, 1286.

[8] Q. Xu , W. Long , H. Jiang , C. Zan , J. Huang , X. Chen , L. Shi , Chem. Eng. J. 2018, 350, 776.

[9] X. Luo , H. Gong , Z. He , P. Zhang , L. He , J. Hazard. Mater. 2020, 399, 123137. 32937726 10.1016/j.jhazmat.2020.123137

[10] P. Cherukupally , W. Sun , A. P. Y. Wong , D. R. Williams , G. A. Ozin , A. M. Bilton , C. B. Park , Nat. Sustain. 2019, 3, 136.

[11] C. Tang , Y. Zhu , H. Bai , G. Li , J. Liu , W. Wu , Y. Yang , S. Xuan , H. Yin , Z. Chen , L. Lai , Y. Song , M. Cao , B. Qiu , ACS Appl. Mater. Interfaces 2023, 15, 49973. 10.1021/acsami.3c10211 37843979

[12] H. Bi , Z. Yin , X. Cao , X. Xie , C. Tan , X. Huang , B. Chen , F. Chen , Q. Yang , X. Bu , X. Lu , L. Sun , H. Zhang , Adv. Mater. 2013, 25, 5916. 24038404 10.1002/adma.201302435

[13] B. Liu , B. Chen , J. Ling , E. J. Matchinski , G. Dong , X. Ye , F. Wu , W. Shen , L. Liu , K. Lee , L. Isaacman , S. Potter , B. Hynes , B. Zhang , J. Hazard. Mater. 2022, 437, 129340. 35728323 10.1016/j.jhazmat.2022.129340

[14] Y. Guo , Z. Guo , W. Liu , Droplet 2023, 2, 75.

[15] S. Wu , H. Yang , G. Xiong , Y. Tian , B. Gong , T. Luo , T. S. Fisher , J. Yan , K. Cen , Z. Bo , K. K. Ostrikov , ACS Nano 2019, 13, 13027. 31660731 10.1021/acsnano.9b05703

[16] Y. Song , L. Shi , H. Xing , K. Jiang , J. Ge , L. Dong , Y. Lu , S. Yu , Adv. Mater. 2021, 33, 2100074. 10.1002/adma.202100074 34297448

[17] Y. Ye , T. Li , Y. Zhao , J. Liu , D. Lu , J. Wang , K. Wang , Y. Zhang , J. Ma , E. Drioli , X. Cheng , Sep. Purif. Technol. 2023, 317, 123885.

[18] X. Cheng , T. Li , L. Yan , Y. Jiao , Y. Zhang , K. Wang , Z. Cheng , J. Ma , L. Shao , Sci. Adv. 2023, 9, adh8195. 10.1126/sciadv.adh8195 PMC10446487 37611103

[19] X.‐Y. Guo , L. Zhao , H.‐N. Li , H.‐C. Yang , J. Wu , H.‐Q. Liang , C. Zhang , Z.‐K. Xu , Science 2024, 386, 654. 39509489 10.1126/science.adq6329

[20] X. Huang , Y. Sun , S. Soh , Adv. Mater. 2015, 27, 4062. 26043083 10.1002/adma.201501578

[21] G. Kwon , D. Panchanathan , S. R. Mahmoudi , M. A. Gondal , G. H. McKinley , K. K. Varanasi , Nat. Commun. 2017, 8, 14968. 28440292 10.1038/ncomms14968 PMC5413974

[22] B. Xin , J. Hao , Chem. Soc. Rev. 2010, 39, 769. 20111792 10.1039/b913622c

[23] J.‐J. Li , Y.‐N. Zhou , Z.‐H. Luo , Prog. Polym. Sci. 2018, 87, 1.

[24] H. Zhang , Z. Guo , Adv. Colloid Interface Sci. 2023, 320, 103003. 37778250 10.1016/j.cis.2023.103003

[25] B. Buszewski , S. Bocian , A. Felinger , Chem. Rev. 2012, 112, 2629. 22309131 10.1021/cr200182j

[26] P. Marchetti , M. F. Jimenez Solomon , G. Szekely , A. G. Livingston , Chem. Rev. 2014, 114, 10735. 25333504 10.1021/cr500006j

[27] Y. Yang , Z. Guo , W. Liu , Small 2022, 18, 2204624. 10.1002/smll.202204624 36192169

[28] B. Wang , W. Liang , Z. Guo , W. Liu , Chem. Soc. Rev. 2015, 44, 336. 25311259 10.1039/c4cs00220b

[29] B. Xiang , Q. Sun , Q. Zhong , P. Mu , J. Li , J. Mater. Chem. A 2022, 10, 20190.

[30] H. Wang , X. Zhao , Z. Xie , B. Yang , J. Zheng , K. Yin , Z. Zhou , Int. J. Extrem. Manuf. 2024, 6, 045503.

[31] Z. Liu , Y. Si , C. Yu , L. Jiang , Z. Dong , Chem. Soc. Rev. 2024, 53, 10012. 39302142 10.1039/d4cs00673a

[32] R. Tao , W. Fang , J. Wu , B. Dou , W. Xu , Z. Zheng , B. Li , Z. Wang , X. Feng , C. Hao , Research 2023, 6, 0023. 37040478 10.34133/research.0023 PMC10076004

[33] J. Sun , X. Qin , Y. Song , Z. Xu , C. Zhang , W. Wang , Z. Wang , B. Wang , Z. Wang , Int. J. Extrem. Manuf. 2023, 5, 025504.

[34] Z. Zhan , Y. Su , M. Xie , Y. Li , Y. Shuai , Z. Wang , Mater. Today 2024, 80, 529.

[35] Z. Wang , Q. Yin , Z. Zhan , W. Li , M. Xie , H. Duan , P. Cheng , C. Zhang , Y. Chen , Z. Dong , Int. J. Extrem. Manuf. 2023, 5, 025502.

[36] Y. Tian , P. Zhu , X. Tang , C. Zhou , J. Wang , T. Kong , M. Xu , L. Wang , Nat. Commun. 2017, 8, 1080. 29057877 10.1038/s41467-017-01157-4 PMC5714965

[37] S. Zhang , M. Chi , J. Mo , T. Liu , Y. Liu , Q. Fu , J. Wang , B. Luo , Y. Qin , S. Wang , S. Nie , Nat. Commun. 2022, 13, 4168. 35851036 10.1038/s41467-022-31987-w PMC9293931

[38] L. Wu , Z. Dong , Z. Cai , T. Ganapathy , N. X. Fang , C. Li , C. Yu , Y. Zhang , Y. Song , Nat. Commun. 2020, 11, 521. 31988314 10.1038/s41467-020-14366-1 PMC6985111

[39] Z. Wang , C. Wu , X. Wang , M. Xie , Y. Li , Z. Zhan , Y. Shuai , Adv. Funct. Mater. 2025, 35, 2416014.

[40] C. Yang , Y. Yu , Y. Zhao , L. Shang , Research 2023, 6, 0034. 37040286 10.34133/research.0034 PMC10076059

[41] C. Yang , Y. Yu , L. Shang , Y. Zhao , Nat. Chem. Eng. 2024, 1, 87.

[42] M. Xie , X. Wang , Z. Qian , Z. Zhan , Q. Xie , X. Wang , Y. Shuai , Z. Wang , Small 2025, 21, 2406844. 10.1002/smll.202406844 39370664

[43] X. Leng , L. Sun , Y. Long , Y. Lu , Droplet 2022, 1, 139.

[44] M. Xie , Z. Zhan , Y. Li , J. Zhao , C. Zhang , Z. Wang , Z. Wang , Int. J. Extrem. Manuf. 2024, 6, 032005.

[45] J. Xu , S. Xiu , Z. Lian , H. Yu , J. Cao , Droplet 2022, 1, 11.

[46] Y. Li , Z. Cui , G. Li , H. Bai , R. Dai , Y. Zhou , Y. Jiao , Y. Song , Y. Yang , S. Liu , M. Cao , Adv. Funct. Mater. 2022, 32, 2201035.

[47] D. Wang , H. Huang , F. Min , Y. Li , W. Zhou , Y. Gao , G. Xie , Z. Huang , Z. Dong , Z. Chu , Small 2024, 20, 2402946. 10.1002/smll.202402946 38881253

[48] G. Li , M. Zhang , S. Liu , M. Yuan , J. Wu , M. Yu , L. Teng , Z. Xu , J. Guo , G. Li , Z. Liu , X. Ma , Nat. Electron. 2023, 6, 154.

[49] H. Xing , X. He , Y. Wang , X. Zhang , L. Li , Y. Wang , Z. Cheng , H. Wu , Q. Ge , X. Li , Mater. Today 2023, 68, 84.

[50] Z. Lian , J. Zhou , W. Ren , F. Chen , J. Xu , Y. Tian , H. Yu , Int. J. Extrem. Manuf. 2024, 6, 012008.

[51] Z. Xue , Y. Cao , N. Liu , L. Feng , L. Jiang , J. Mater. Chem.A 2014, 2, 2445.

[52] R. K. Gupta , G. J. Dunderdale , M. W. England , A. Hozumi , J. Mater. Chem.A 2017, 5, 16025.

[53] Z. Chu , Y. Feng , S. Seeger , Angew. Chem., Int. Ed. 2015, 54, 2328. 10.1002/anie.201405785 25425089

[54] X. Zhou , Z. Zhang , X. Xu , F. Guo , X. Zhu , X. Men , B. Ge , ACS Appl. Mater. Interfaces 2013, 5, 7214. 10.1021/am4015346 23823678

[55] A. K. Singh , J. K. Singh , RSC Adv. 2016, 6, 103632.

[56] S. Oh , S. Ki , S. Ryu , M. C. Shin , J. Lee , C. Lee , Y. Nam , Langmuir 2019, 35, 7769. 31099245 10.1021/acs.langmuir.9b00993

[57] X. Liu , L. Ge , W. Li , X. Wang , F. Li , ACS Appl. Mater. Interfaces 2015, 7, 800. 10.1021/am507238y 25490110

