# Supplementary material for: Biomimetic Microfluidic Pumps for Selective Oil–Water Separation
Source: Adv Sci (Weinh). 2025 Apr 26;12(27):2503511. doi: 10.1002/advs.202503511 (PMC12279168; doi:10.1002/advs.202503511)
Supplement: Supplementary file 1 — Supporting Information [file ADVS-12-2503511-s005.docx]

Supporting Information

Biomimetic microfluidic pumps for selective oil–water separation

Zhaolong Wang*, Yinfeng Li, Mingzhu Xie, Ziheng Zhan, Wenhao Li, Qihui Xie, Yong Shuai*, Zhichao Dong*, Zuankai Wang*

Prof. Z. Wang, Y. Li, M. Xie, Z. Zhan, W. Li, Q. Xie, Prof. Y. Shuai

School of Energy Science and Engineering

Harbin Institute of Technology

Harbin 150001, P. R. China

E-mail: wangzhaolong@hit.edu.cn; shuaiyong@hit.edu.cn

Prof. Z. Wang, Y. Li, W. Li, Prof. Y. Shuai

Zhengzhou Research Institute

Harbin Institute of Technology

Zhengzhou 450046, P. R. China

Prof. Z. Dong

Technical Institute of Physics and Chemistry

Chinese Academy of Sciences

Beijing 100190, P. R. China

E-mail: dongzhichao@mail.ipc.ac.cn

Prof. Z. Wang

Department of Mechanical Engineering

Hong Kong Polytechnic University

Hong Kong 999077, P. R. China

E-mail: zk.wang@polyu.edu.hk

**Supplementary Figures**

**
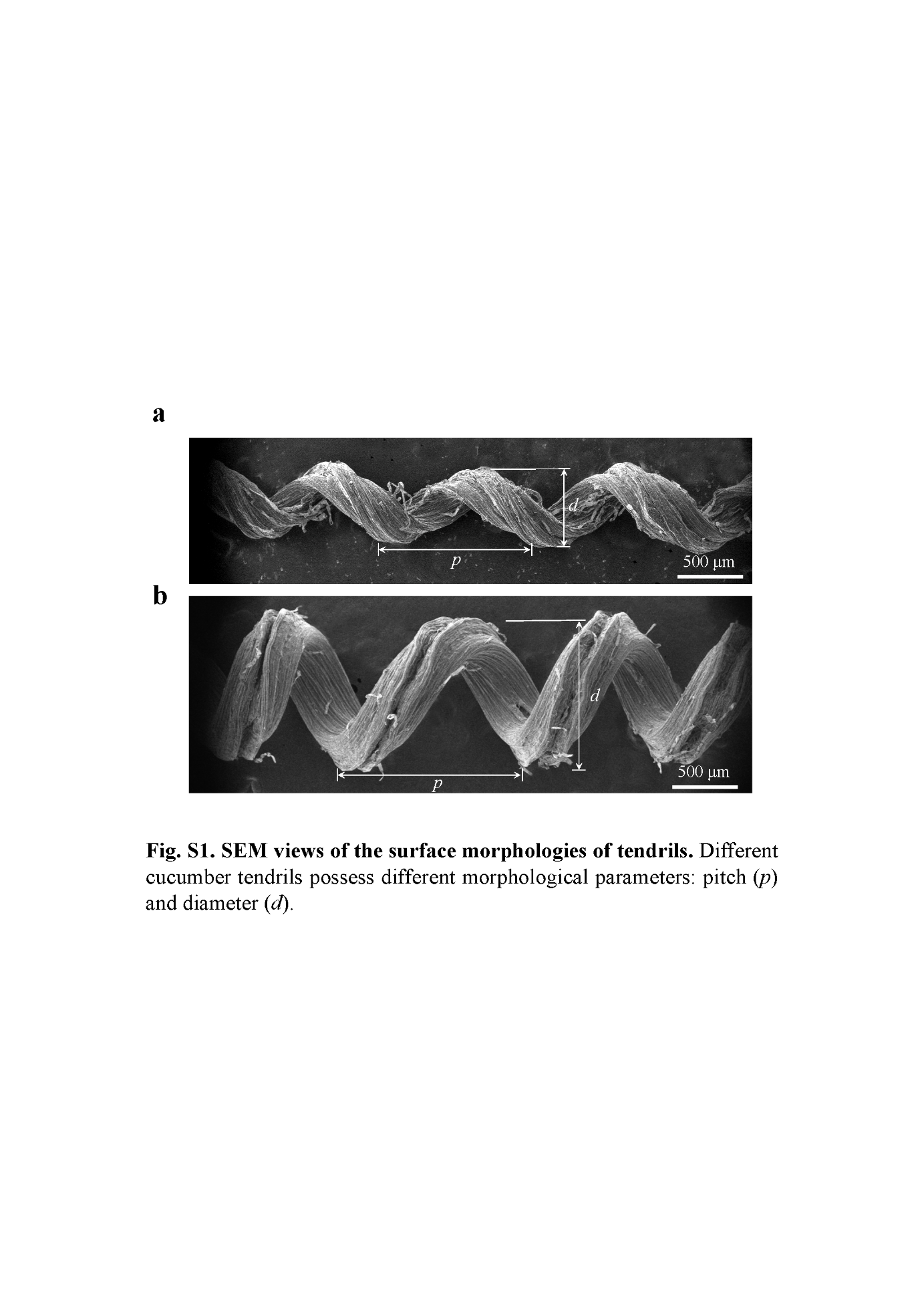
**

**Figure S1.** SEM views of the surface morphologies of tendrils. Different cucumber tendrils possess different morphological parameters: pitch (*p*) and diameter (*d*).


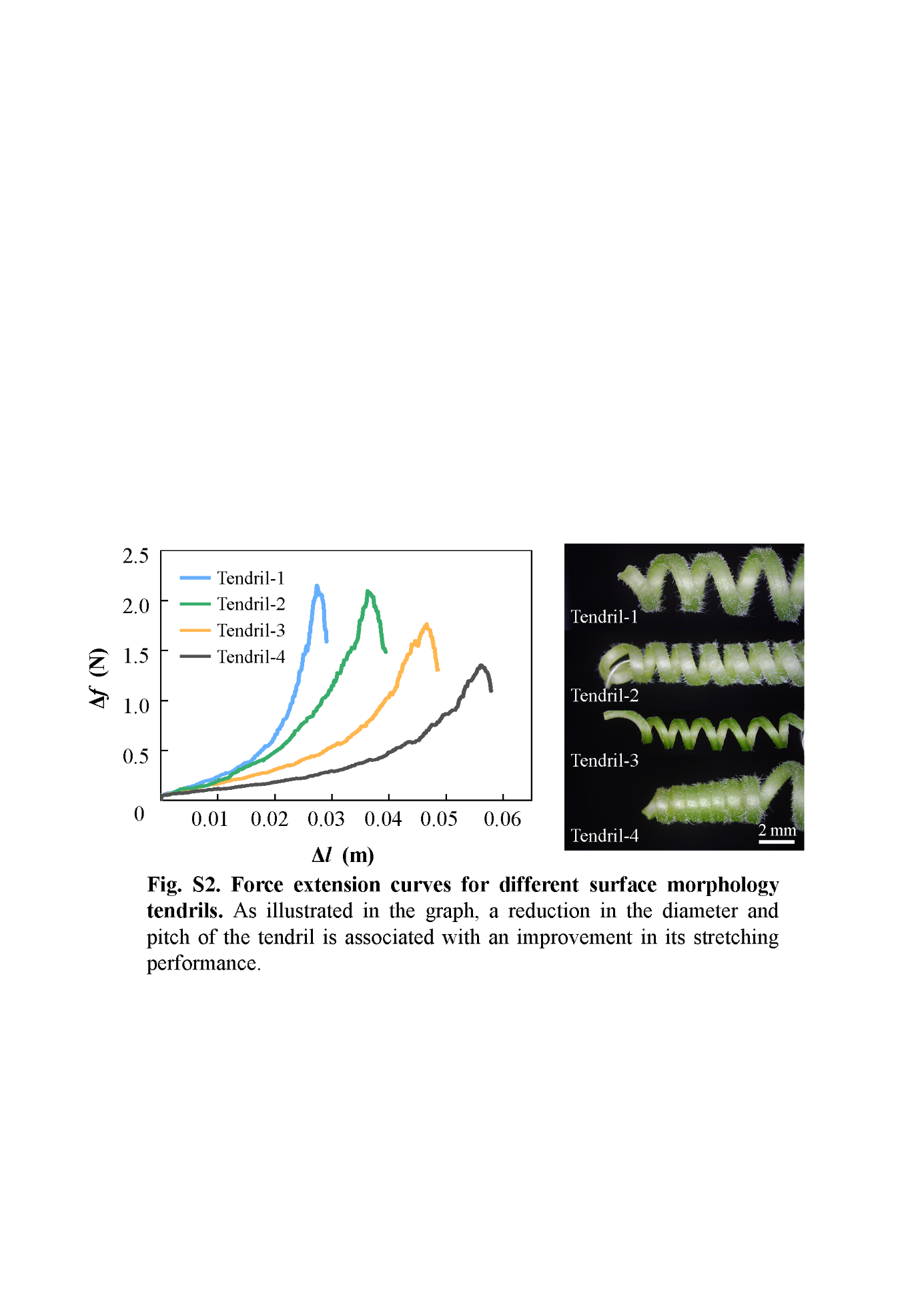


**Figure S2.** Force extension curves for different surface morphology tendrils. As illustrated in the graph, a reduction in the diameter and pitch of the tendril is associated with an improvement in its stretching performance.


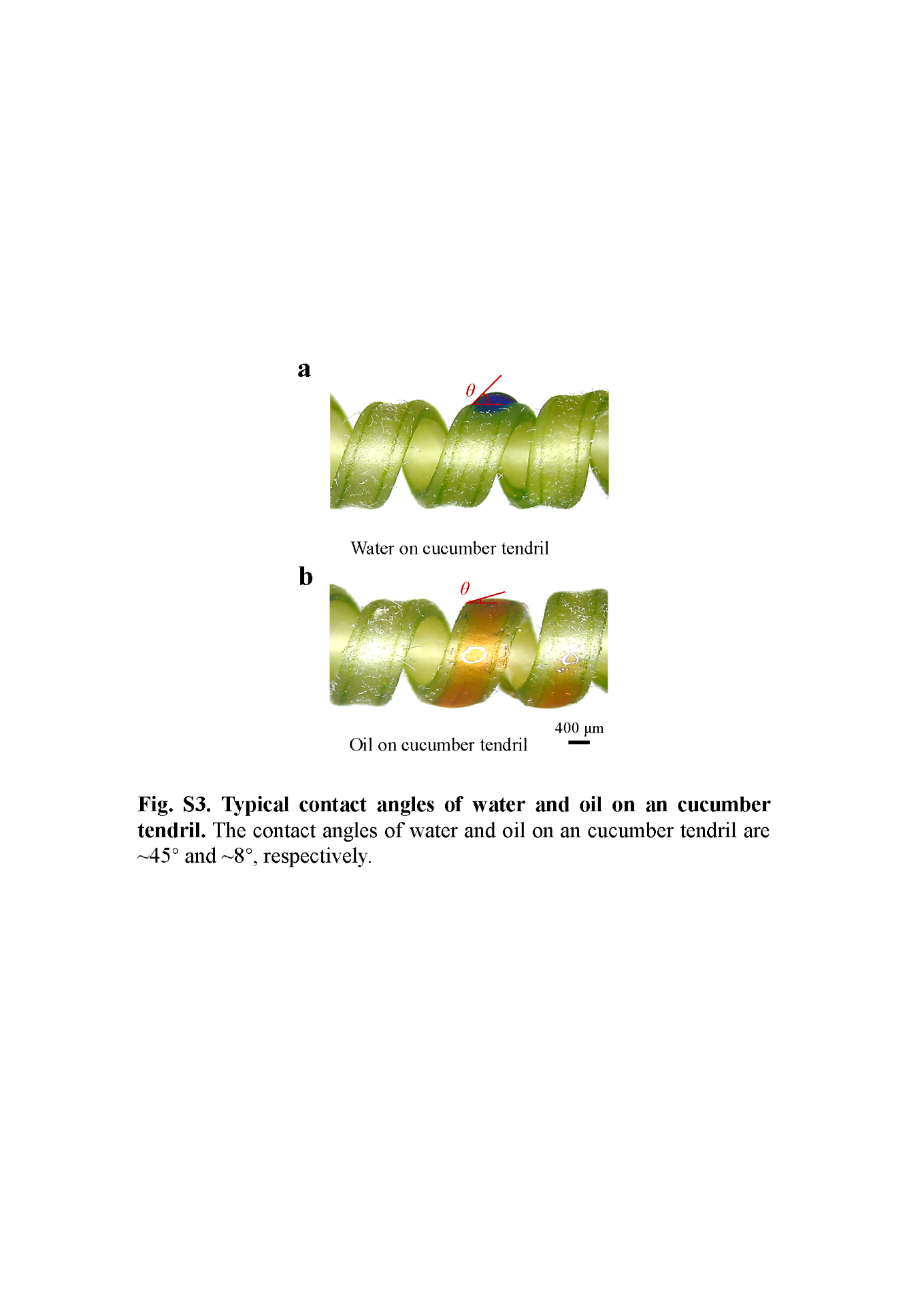


**Figure S3.** Typical contact angles of water and oil on a cucumber tendril. The contact angles of water and oil on a cucumber tendril are ~45° and ~8°, respectively.

**Figure S4.** The fabricated biomimetic spring microchannel (SMC) via PμSL based 3D printing technology.


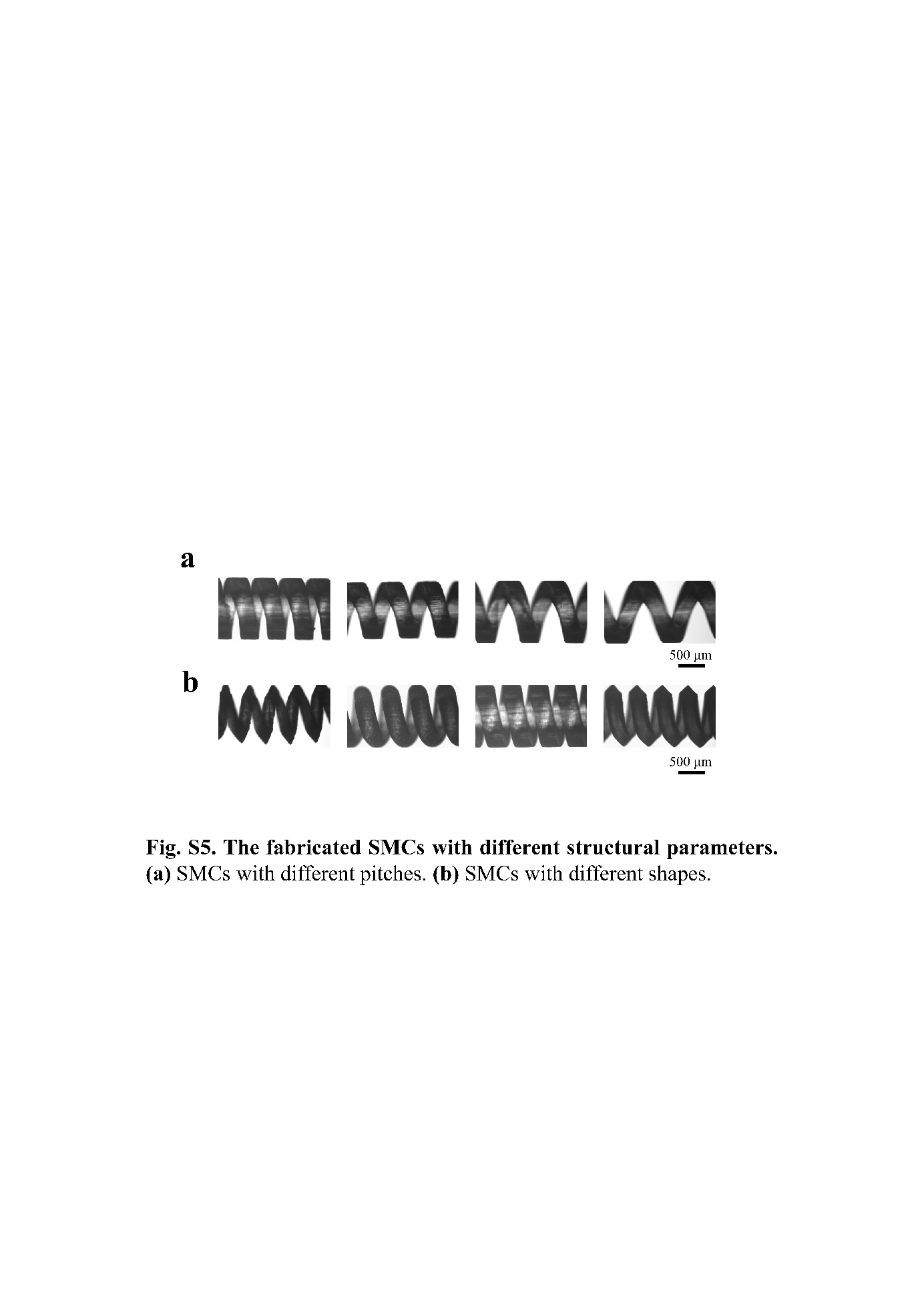


**Figure S5.** The fabricated SMCs with different structural parameters. a) SMCs with different pitches. b) SMCs with different shapes.


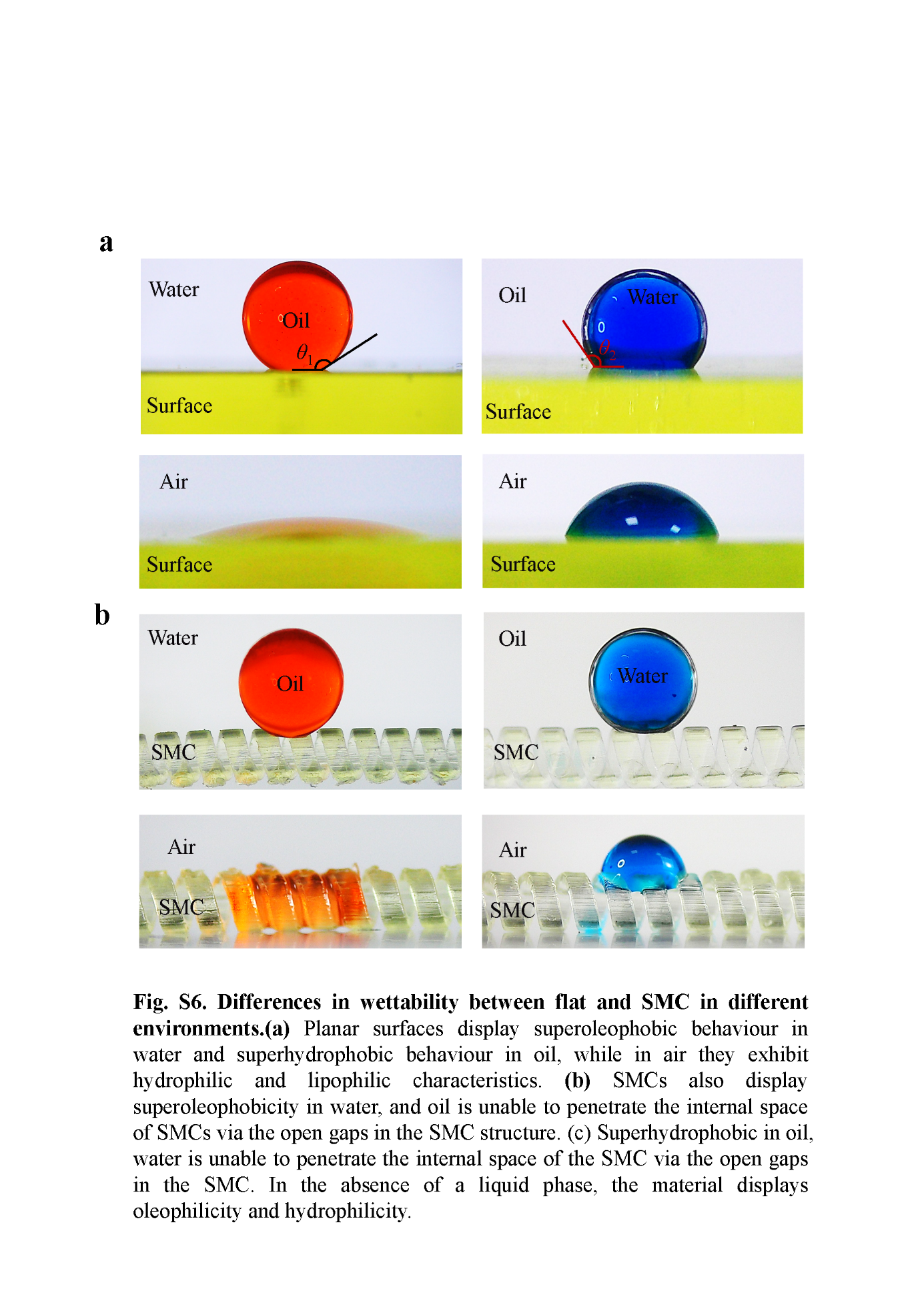


**Figure S6.** Differences in wettability between flat and SMC in different environments. a) Planar surfaces display superoleophobic behavior in water and superhydrophobic behavior in oil, while they exhibit hydrophilic and lipophilic characteristics in air. b) SMCs also display superoleophobicity in water, and oil is unable to penetrate the internal space of SMCs via the open gaps in the SMC structures. SMCs display superhydrophobic in oil, water is unable to penetrate the internal space of the SMC via the open gaps in the SMC. In the absence of a liquid phase, the material displays oleophilicity and hydrophilicity.

**Figure S7.** Differences in wettability between flat and SMC in different environments. a) and b) are the schematic diagram of the separation and the actual optical microscope picture, respectively. When the SMC filled with water in advance and placed at the oil-water interface, due to the difference in molecular polarity and surface wettability, the oil cannot enter into the SMC while the water can flow in the SMC. In contrast, when the SMC capillary filled with oil in advance and placed at the oil-water interface, the water cannot enter into the SMC and the oil can flow in the SMC.

**Figure S8.** Controlled transport of SMC stretched fluids. When SMC is stretched, the change in diameter and tooth pitch will result in a change in the capillary drive force, which allows for the tailored transport of the fluid over the distance.

**Figure S9.** The *h*-*t* diagrams of water transport in SMCs with different structural parameters. a) SMCs with different diameters. b) SMCs with different pitches.

**Figure S10.** The advancing CA of printed flat surfaces with different treatments. a) SEM images of non-treated planar and TiO_2_ nanoparticle-coated planar surfaces. b) The advancing CA plays a key role in the rising height where the capillary force, viscous force, and gravity of the liquid are in equilibrium. Thus, we compare the advancing CA of printed flat surfaces with different treatments including TiO_2_ nanoparticle, plasma treatment, and no treatment，whose advancing CAs are 86°, 18° and 38°, respectively.

**Figure S11.** Meniscus formed with liquids in SMC and the dynamic evolution of curvature radius and Laplacian pressure difference of concave surface during SMC stretching and curving.

**Figure S12.** Liquid pumping and force analysis in SMCs. The forces acting on the liquid of the SMC during separation can be divided into four main categories: (i) pump pressure, (ii) liquid surface tension, (iii) liquid gravity, and (iv) adhesive force.

**Figure S13.** Stress-strain curves of SMC under unwetted condition.

**Figure S14.** The surface wettability of different liquids.

**Figure S15.** The crude oil separation performance of SMC separation system. As illustrated in the graph, crude oil volume changes in the syringe during crude oil separation.

**Figure S16.** Separation of crude oil by multiple SMCs at the same time.
